# Whole-genome resequencing of *Escherichia coli *K-12 MG1655 undergoing short-term laboratory evolution in lactate minimal media reveals flexible selection of adaptive mutations

**DOI:** 10.1186/gb-2009-10-10-r118

**Published:** 2009-10-22

**Authors:** Tom M Conrad, Andrew R Joyce, M Kenyon Applebee, Christian L Barrett, Bin Xie, Yuan Gao, Bernhard Ø Palsson

**Affiliations:** 1 Department of Chemistry and Biochemistry, University of California San Diego, 9500 Gilman Drive, La Jolla, California, 92093-0332, USA; 2 Department of Bioengineering, University of California San Diego, 9500 Gilman Drive, La Jolla, California, 92093-0412, USA; 3 Department of Computer Science, Virginia Commonwealth University, 401 West Main Street, Richmond, Virginia, 23284-3019, USA; 4 Center for the Study of Biological Complexity, Virginia Commonwealth University, 1000 W. Cary St., Richmond, Virginia, 23284-3068, USA

## Abstract

**Background:**

Short-term laboratory evolution of bacteria followed by genomic sequencing provides insight into the mechanism of adaptive evolution, such as the number of mutations needed for adaptation, genotype-phenotype relationships, and the reproducibility of adaptive outcomes.

**Results:**

In the present study, we describe the genome sequencing of 11 endpoints of *Escherichia coli *that underwent 60-day laboratory adaptive evolution under growth rate selection pressure in lactate minimal media. Two to eight mutations were identified per endpoint. Generally, each endpoint acquired mutations to different genes. The most notable exception was an 82 base-pair deletion in the *rph*-*pyrE *operon that appeared in 7 of the 11 adapted strains. This mutation conferred an approximately 15% increase to the growth rate when experimentally introduced to the wild-type background and resulted in an approximately 30% increase to growth rate when introduced to a background already harboring two adaptive mutations. Additionally, most endpoints had a mutation in a regulatory gene (*crp *or *relA*, for example) or the RNA polymerase.

**Conclusions:**

The 82 base-pair deletion found in the *rph*-*pyrE *operon of many endpoints may function to relieve a pyrimidine biosynthesis defect present in MG1655. In contrast, a variety of regulators acquire mutations in the different endpoints, suggesting flexibility in overcoming regulatory challenges in the adaptation.

## Abstract 2

Escherichia coli strains that have evolved in the laboratory in response to lactate minimal media show a wide range of different genetic adaptations.

## Background

One hundred and fifty years after the publication of *The Origin of Species*, evolution is still a topic of great interest for researchers today due in large part to advances in DNA sequencing technology. *De novo *genomic sequencing is being carried out on a massive scale and large databases of biological sequence data, such as the NCBI Entrez Genome Project [1] and Genomes OnLine Database (GOLD) [2], are constantly expanding. This genomic information has been interrogated using comparative genomics to infer evolutionary histories and basic principles of evolution in bacteria (see [3] for a review). While a wealth of knowledge has been learned from these studies, they are usually coarse-grained, focusing on gene loss, horizontal gene transfer, and general statistics of sequence changes. The importance of individual single nucleotide polymorphisms (SNPs) and small insertions/deletions (indels) when comparing divergent strains is difficult to determine using comparative genomics because these changes occur with high frequency and are often selectively neutral, necessitating intensive use of population genetics to distinguish selective mutations [4].

More recently, platforms allowing a base-by-base comparison between highly similar genomes have been developed [5,6]. Such technology can now be utilized to perform before-and-after experiments, where the genetic changes in a population occurring during real time are measured. This advance allows the unprecedented ability to observe the genetic basis of adaptive evolution directly, rather than through inference of evolutionary histories. Additionally, these studies allow the contribution of mutations to adaptation to be observed clearly.

Owing to short generation times, large population sizes, repeatability, and the ability to preserve ancestor strains by freezing for later direct comparison of distant generations, microorganisms have been used to study adaptive evolution [7]. Whole-genome resequencing of microorganisms following adaptive evolution has the potential to discover fundamental parameters of adaptive evolution in bacteria, including the number of mutations acquired during adaptation, functions of the mutated genes, and repeatability of the genetic changes in replicate experiments. However, presently only a small number of studies of adaptive evolution in bacteria have included resequencing of the genome [8-10]. One such study included the resequencing of yeast evolved to glucose, phosphate, or sulfate limitation in a chemostat [11]. While yeast was constrained in which genes mutated in the sulfate-limited condition due to a single optimal adaptive solution to the condition, glucose- and phosphate- limited conditions had a number of equivalent solutions to the condition and so more variability in observed mutations was observed. Their work suggests that the parameters of adaptive evolution vary with condition.

We previously reported the sequencing of *E. coli *following short-term (approximately 40 days) adaptive evolution in glycerol minimal media to obtain its computationally predicted phenotype [10]. The number and location of genes was highly similar among replicates, with mutations in the glycerol kinase and RNA polymerase genes present in most evolved strains. Experiments showed that a single mutation in glycerol kinase or RNA polymerase genes could account for up to 60% of the adaptive improvement in growth phenotype. However, because adaptive evolution in only a single condition was studied, it is not clear whether findings, such as the number, consistency, and impact of mutations, are typical for short-term adaptive evolution of *E*. *coli *in minimal media.

*E. coli *K-12 MG1655 that has undergone adaptation in lactate M9 minimal media shows fitness gains of a magnitude similar to those observed in glycerol M9 minimal media [12]. Herein we describe analogous experiments detailing the sequencing of *E. coli *adaptively evolved in lactate minimal media, and the fitness benefits of the discovered mutations. We found that changing the carbon source affects adaptive parameters, including the number of mutations needed for adaptation and the diversity of genotypic outcomes.

## Results and discussion

### Comparative genome sequencing

Five parallel adaptive evolutions of *E. coli *MG1655 (LactA, LactB, LactC, LactD, and LactE) over 60 days (approximately 1,100 generations) [12], and later six additional adaptive evolutions (LactF, LactG, LactH, LactI, LactJ, and LactK) over 50 days (approximately 750 generations), were carried out using continuous exponential growth in 2 g/L L-lactate M9 minimal media at 30°C, resulting in an average 90% increase in the growth rate versus the starting strain. To determine the genetic mechanism of adaptation in these strains, the genomes of single colonies from each endpoint culture were sequenced using Nimblegen Comparative Genome Sequencing (CGS) [5] and later 1G Solexa or 2G Solexa sequencing. Comprehensive lists of mutations reported using Nimblegen and Solexa sequencing are included as Additional data files 1 and 2. Regardless of the sequencing method, reported mutations were tested for actual presence in the endpoint colony using Sanger sequencing. The confirmed mutations are shown in Table 1.

**Table 1:** Confirmed mutations discovered in eleven endpoint strains of MG1655 adapted to growth in lactate minimal media

Endpoint	Gene	Product/duplication	Class	Nucleotide	Codon	Protein change
LactA	*crp*	cAMP response protein	Regulator	t452a	CTG->CAG	L151Q
	*hfq*	RNA binding protein	Regulator	c28t	CCG->TCG	P10S
	*ydjO*	Predicted protein	-	t138g	GGT->GGG	G46G
		~87 kb duplication (3946000-4033000)				
						
LactB	*gcvT*	Glycine cleavage system	Metabolic	Δ1 bp (971)	Frameshift	
		~44 kb duplication (1248300-1292200)				
						
LactC	*rph-pyrE*	RNase PH/orotate phosphoribosyltransferase	Metabolic	Δ82bp	Frameshift	
	*cya*	Adenylate cyclase	Regulator	c547t	CTT->TTT	L183F
	*infC*	IF-3	Translation	g283a	GAA->AAA	E95K
						
LactD	*rph-pyrE*	RNase PH/orotate phosphoribosyltransferase	Metabolic	Δ82 bp	Frameshift	
	*ppsA*	Phosphoenolpyruvate synthase	Metabolic	c288a	ATC->ATA	I96I
	*atoS*	AtoS/AtoC two component regulatory system	Regulator	a1367c	CAA->CCA	Q456P
	*relA*	ppGpp synthetase	Regulator	a956c	TAT->TCT	Y319S
	*rho*	Transcription termination factor	Regulator	c304t	CGC->TGC	R102C
	*hepA*	RNAP recycling factor	Regulator	c2665t	CAA->TAA	Q889(stop)
	*kdtA*	KDO transferase	Cell envlp.	t701a	GTA->GAA	V234E
						
LactE	*ppsA*	Phosphoenolpyruvate synthase	Metabolic	c17t	TCG->TTG	S6L
	*acpP*	Acyl carrier protein	Metabolic	g50t	GGC->GTC	G17V
	*hfq*	RNA binding protein	Regulator	c28t	CCG->TCG	P10S
	*crp*	cAMP response protein	Regulator	t497c	ATC->ACC	I166T
	*ydcI*	Putative transcriptional regulator	-	g41a	CGC->CAC	R14H
	*yjbM*	Predicted protein	-	g141a	ATG->ATA	M47I
		~140 kb duplication (3620000-3760000), ~87 kb duplication (3946000-4033000)				
						
LactF	*rph-pyrE*	RNase PH/orotate phosphoribosyltransferase	Metabolic	Δ82 bp	Frameshift	
	*kdtA*	KDO transferase	Cell envlp.	g292a	GGG->AGG	G98R
	*rpoC*	RNA polymerase	Regulator	c2524t	CGT->TGT	R842C
	*argS*	Arginyl-tRNA synthetase	Translation	g110c	GGC->GCC	G37A
		~12 kb duplication (1774000-1786000)				
						
LactG	*rph-pyrE*	RNase PH/orotate phosphoribosyltransferase	Metabolic	Δ82 bp	Frameshift	
	*trpB*	Tryptophan synthase	Metabolic	g462t	GCG->GCT	A154A
	*nadB*	NAD biosynthesis	Metabolic	c405t	GCC->GCT	A135A
	*rpoB*	RNA polymerase	Regulator	a1664c	TAC->TCC	Y555S
	*rpoS*	σ^S^	Regulator	Δ1 bp (609)	Frameshift	
	*kdtA*	KDO transferase	Cell envlp.	g292a	GGG->AGG	G98R
	*osmF*	ABC transporter involved in osmoprotection	Cell envlp.	ins T after 873	AAA->TAA	K292(stop)
	*proQ*	Predicted structural transport element	Cell envlp.	g(-8)t	Promoter	
						
LactH	*rph-pyrE*	RNase PH/orotate phosphoribosyltransferase	Metabolic	Δ82 bp	Frameshift	
	*pdxB*	Erythronate-4-phosphate dehydrogenase	Metabolic	g286t	GTG->TTG	V96L
	*ilvG_1*	Acetolactate synthase II (pseudogene)	Metabolic	Δ1 bp (977)	Frameshift	
	*rpoB*	RNA polymerase	Regulator	Δ1 bp (4006)	Frameshift	
	*kdtA*	KDO transferase	Cell envlp.	g292a	GGG->AGG	G98R
	*wcaA*	Glycosyl transferase	Cell envlp.	Δ4 bp (506509)	Frameshift	
						
LactI	*rph-pyrE*	RNase PH/orotate phosphoribosyltransferase	Metabolic	Δ82 bp	Frameshift	
	*relA*	ppGpp synthetase	Regulator	g4c	GTT->CTT	V2L
	*proQ*	Predicted structural transport element	Cell envlp.	ins T after 15	Frameshift, AAG->TAA	K6(stop)
						
LactJ	*rph-pyrE*	RNase PH/orotate phosphoribosyltransferase	Metabolic	Δ82 bp	Frameshift	
	*mrdA*	Peptidoglycan synthetase, PBP2	Cell envlp.	c157a	CGC->AGC	R53S
	*rpsA*	30S ribosomal subunit	Translation	a490t	AAC->TAC	N164Y
	*kgtP*	Á-ketoglutarate MFS transporter	Cell envlp.	g1083a	AAG->AAA	K361K
	*kgtP*			Δ1 bp (1212)	Frameshift	
		Intergenic		g3630812t		
						
LactK	*ppsA*	Phosphoenolpyruvate synthase	Metabolic	g61a	GTA->ATA	V21I
	*rpoC*	RNA polymerase	Regulator	Δ9 bp (36113619)	In frame	V1204G
	*ryhA*	Small RNA that interacts with Hfq	Regulator	c(-9)t	Promoter	
	*treA*	Trehalase	Osmotic	g676a	GCG->ACG	A226T
	*secE*	Sec protein secretion complex	Cell envlp.	g350a	CGC->CAC	R117H
	*secF*	Sec protein secretion complex	Cell envlp.	g109a	GCT->ACT	A37T
		~40 kb duplication (1253000-1294000)				

DNA from single colonies isolated from the endpoints of the 11 strains adapted to growth on lactate M9 minimal media were screened for mutations using Nimblegen CGS and Solexa technologies. Mutations (except for large duplications) were confirmed by Sanger sequencing of the DNA isolated from the single colonies using primers flanking the mutated site. Nucleotide changes refer to position within the respective gene, deletions are indicated by the Δ symbol, and insertions are marked by 'ins'. The *rph*-*pyrE *Δ82 bp mutation is described in Figure 3. Genomic coordinates of large duplications are shown in parentheses. Cell envlp., cell envelope.

Nimblegen CGS has been used previously to identify the SNPs, deletions, and duplications acquired by bacteria during adaptive evolution [10]. This approach is based on the decreased hybridization of mutated DNA to corresponding probes in genomic tiling arrays relative to hybridization of non-mutated DNA. In this study, CGS identified a total of 93 mutations in five evolved strains (LactA to LactE). Of these, we found 14 confirmed SNPs and 67 false positives. Twenty-two reported SNPs were actually discrepancies between the sequences of MG1655 used to create the tiling arrays and the MG1655 strain used to begin the adaptive evolutions. The observed false positive rate (1 per 340,000 bp) is highly similar to the rate previously observed [10] for CGS.

We later attempted sequencing of the endpoint strains using G1 Solexa (LactA, LactB, LactC, and LactE), and then G2 Solexa (LactB, LactD, LactF to LactK). Instead of measuring DNA hybridization, Solexa relies on the generation of short sequence reads through reverse-termination synthesis. The reads are mapped onto a reference genome, and consistent non-exact matches are reported as mutations. G1 Solexa succeeded in detecting several mutations in LactA and LactE missed by analysis of CGS data for these strains. However, depending on the mapping technique and stringency used for reporting mutations, analysis of G1 Solexa data resulted in either many false negatives or many false positives. When sequencing by G2 Solexa became available, the average coverage of sequenced strains greatly improved from 10× coverage using G1 Solexa to more than 40×. The high coverage of reads generated by G2 Solexa resulted in a false positive rate of only one false positive per 9,200,000 bp.

Analysis of G2 Solexa data from 8 endpoint strains resulted in the confirmation of 30 SNPs, 14 deletions, and 3 insertions, in total. Based on a low calculated false negative rate (1 to 2%) for SNPs and deletions (Additional data file 3; see Materials and methods for details), it is very unlikely that more than a few of these types of mutations were not identified in strains sequenced using G2 Solexa. However, detection of small insertions (1 to 4 bp) was less consistent (13% false negative rate) than detection of SNPs and deletions, and larger insertions were not generally detectable by our methods. Therefore, it remains a possibility that several insertions are currently left undetected in these strains.

Additionally, while Solexa sequencing is an excellent tool for determining SNPs and deletions on the genome scale in bacteria, it has the disadvantage that locations of duplicated genome segments and chromosomal rearrangements cannot be determined due to short read length. Pulse field gel electrophoresis [13] or sequencing using longer read lengths, such as 454 [14], or paired reads can provide information on these mutation events. Because these methods are not included in our study, it must be kept in mind that genomic rearrangements may have occurred, but cannot be observed. Despite these shortcomings, approximately five mutations were detected per endpoint strain, and we believe these are informative for the process of adaptive evolution occurring in these cultures.

### Summary of mutations found

Accounting for SNPs, deletions, and insertions, we found a total of 53 mutations across 11 lactate-evolved strains. The number of mutations found in adapted strains was between two and eight. Approximately two-thirds of discovered mutations were SNPs. These were mostly found within the coding region, with only two cases (*proQ *and *ryhA*) where SNPs were found in a promoter region and one case where a mutation was found in a non-promoter intergenic region. Although most SNPs resulted in an amino acid substitution, 4 of 36 SNPs in the dataset were so-called silent mutations. The indels identified by resequencing were located in coding regions and, except for a 9-bp deletion in the *rpoC *gene of LactK, were out of frame.

**Figure 1:**
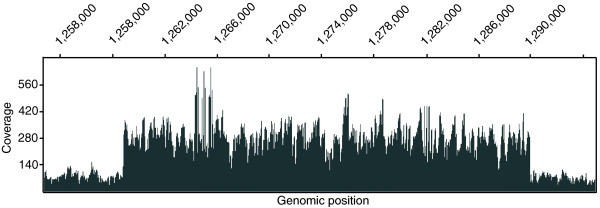
Large genomic duplications. By viewing the coverage of mapped Solexa data graphically across all genomic coordinates, four large duplications were found in the lactate endpoints, two of which are present in two endpoints. The image shows the coverage of mapped Solexa reads from LactK in the region of a large duplication. In total, the following duplications were found: in LactB and LactK, a 4× and 3× duplication of approximately 40 kb from genomic coordinates 1253000 to 1294000; in LactF, a 3× duplication of approximately 12 kb from 1774000 to 1786000; in LactE, a 2× duplication of approximately 140 kb from 3620000 to 3760000; in LactA and LactE, a 2× duplication of approximately 87 kb from 3946000 to 4033000.

Sequencing using Solexa suggested the existence of genomic duplications in several endpoint strains. Data for these strains indicated certain genomic regions that had a higher coverage of mapped reads than the rest of the genome (Figure 1). The increased fold coverage in these regions was calculated across all strains as average coverage across the region divided by average coverage across the genome. Some strains had regions with two- to four-fold coverage, and this was considered indicative of duplication when most other strains had 0.9- to 1.1-fold coverage in the same region (if these regions represented experimental or mapping issues, the enriched coverage regions would have been seen in all strains). We found a total of four regions that were duplicated in at least one adaptive endpoint. The duplications are described in Table 1. Notably, the duplication in LactF doubled the copy number of the *ppsA *gene, which was mutated in three evolved strains (LactD, LactE, LactK). The change in expression levels of genes in these regions due to increased copy number may provide some competitive advantage to the strains, as was observed previously in *Salmonella typhimurium *adapted to limiting amounts of various carbon sources [15].

### Functions of mutated genes

**Figure 2:**
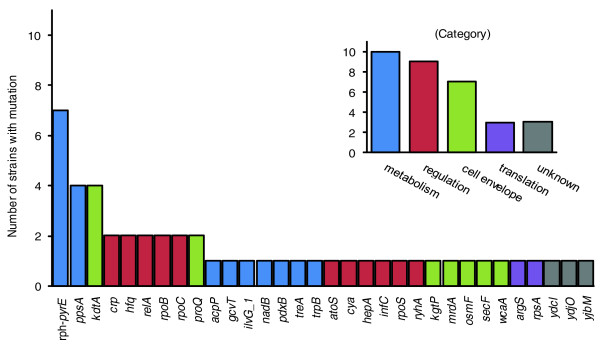
Frequency of mutations. The main graph shows the number of endpoint strains in which a specific gene was mutated out of the 11 adaptive endpoints. The smaller graph shows the number of endpoint strains that have acquired a mutation in at least one gene of a general category, such as metabolism or the cell envelope. The bar color of specific genes in the main graph corresponds to the gene's category classification in the smaller graph.

Mutations affected many different genes with a broad range of cellular functions, but the majority of mutations belong to genes with primary functions relating to metabolism, regulation, or the cell envelope (Figure 2).

**Figure 3:**
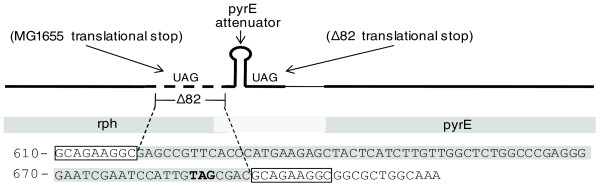
The *rph*-*pyrE *Δ82-bp mutation. An 82-bp deletion in the *rph*-*pyrE *operon was found in 7 of 11 lactate adapted strains. The mutation maps to the end of the *rph *gene, just before the *pyrE *attenuator loop, causing the translational stop codon (TAG, shown in bold) to move from some distance upstream of the attenuator to just downstream of the loop, likely relieving repression of *pyrE *by the attenuator. The sequence in and around the deleted region of the operon is shown. The sequence of the deleted region is shown as highlighted, while a 10-bp sequence that repeats after 82 bp is surrounded with a box. The repeating sequence may explain the frequent occurrence of the deletion as a result of DNA polymerase slippage during DNA replication [27].

The most frequently mutated metabolic genes were *ppsA *and *rph*-*pyrE*. The *E*. *coli *MG1655 laboratory strain used for adaptive evolution has a defect in pyrimidine biosynthesis caused by a 1-bp deletion in the *rph*-*pyrE *operon that results in low levels of orotate phosphoribosyltransferase encoded by *pyrE *[16]. The recurring deletion in *rph*-*pyrE *extends past the 3' end of the *rph *gene, to a region of the operon that is close to an attenuator loop (Figure 3). The deletion shifts the stop codon of the *rph *gene closer to the attenuator loop through a frameshift. Previous experiments suggest that, due to links between translation and the attenuation before transcription of the *pyrE *gene, proper regulation of *pyrE *expression by intracellular uracil levels is achieved by moving the MG1655 *rph *stop codon closer to the attenuator loop [17]. Thus, mutation of the regulatory structure could function to increase orotate phosphoribosyltransferase toward normal levels [16]. However, although the nature of the mutation clearly suggests such a mechanism, previously determined gene expression data did not show significant upregulation of *pyrE *gene expression in the LactC and LactD strains, which harbored the *rph*-*pyrE *deletion. More experiments are needed to conclude an adaptive mechanism for the *rph*-*pyrE *mutations.

The *ppsA *gene encodes the gluconeogenic phosphoenolpyruvate synthase protein and was mutated in four endpoint strains, including a duplication. Gene expression studies indicated *ppsA *was consistently upregulated in lactate-adapted endpoints relative to the pre-evolved MG1655 strain [12]. *In vitro *kinetic assays of phosphoenolpyruvate synthase and quantification of the *ppsA *transcript in the *ppsA *site-directed mutants, including a mutant with a synonymous substitution (silent mutation), indicated that the mutations cause increased expression of *ppsA *rather than altered enzyme kinetics [18]. Recent evidence shows that symonymous mutations can result in drastic changes in expression levels of the gene [19]. Upregulation of *ppsA *expression through mutations to the *ppsA *gene or other means may be of key importance for growth of MG1655 on lactate due to the need for gluconeogenesis to produce biomass precursors.

A diverse set of regulatory genes acquired mutations, including *cyaA*, *crp*, *hfq*, *relA*, *rpoS*, and *ryhA*. The *cyaA *and *crp *genes encode the key proteins for catabolite repression, adenylate cyclase and catabolism repressor protein. A direct relationship also exists between the *hfq *and *ryhA *genes; *ryhA *codes for a small RNA that interacts with *hfq *and may provide regulation [20]. The *relA *gene product synthesizes ppGpp in response to low levels of amino acids, initiating a stringent response [21]. A mutation was found in *rpoS*, the gene encoding the σ^s ^sigma factor responsible for the general stress response and transition to stationary phase. Interestingly, *crp*, *relA*, and *hfq *have also been shown to regulate σ^s ^levels [21-23], suggesting that controlling σ^s ^levels may be a common consequence of the different regulatory mutations. Statistically significant enrichment for downregulation of genes in the σ^s ^regulon in four of five endpoint strains with expression profiles further suggests that countering the stress response is important for adaptation of MG1655 to lactate minimal media [18] (for a complete list of enriched regulons, see Additional data file 4). Alternatively, the variability of differential expression patterns seen in this same dataset also suggests there may be several adaptive ways for MG1655 to alter its transcription state, and downregulation of the stress response may be a common indirect consequence of other adaptive changes to the expression network driven by mutation to various regulatory genes.

In addition to those mutations affecting metabolism and regulation, there are many mutations affecting the cell envelope, such as those in *kdtA *(mutated in four endpoints), which is involved in lipopolysaccharide synthesis, and those in *proQ *and *secF*, which have roles in transport of membrane proteins. The cell envelope provides *E. coli *with an interface to its environment, and previous work has shown the importance of changes to the cell envelope in adaptive evolution of *E. coli *[24]. However, we are unable to infer specific functions of mutations to these genes.

### Time of appearance of acquired mutations

**Figure 4:**
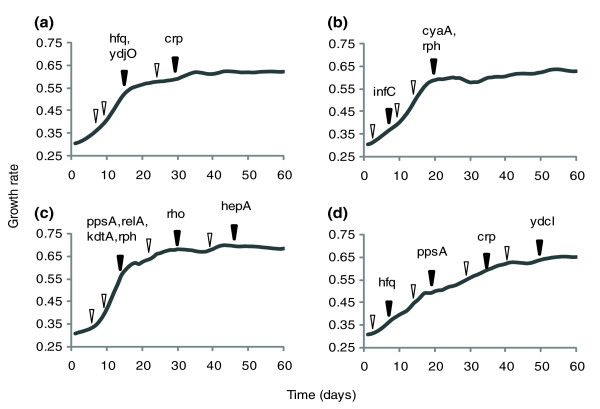
Temporal order of acquired mutations. DNA extracted from frozen intermediate time points of the adaptive evolutions was Sanger sequenced at genomic locations corresponding to mutations in the endpoints. Time points that were sequenced for mutations are indicated by an arrowhead. The arrow is white if no mutations were identified that were not identified at a previous time point. The first day each mutation was observed is indicated with a dark arrow. Curves represent the growth rate trajectory during the period of adaptive evolution. **(a) **LactA, **(b) **LactC, **(c) **LactD, **(d) **LactE. The *atoS*, *acpP*, and *yjbM *genes are not represented in the figure because they were not identified as penetrating more than 50% of the population by day 60 of adaptive evolution.

In order to determine the approximate time of appearance of each mutation in LactA, LactC, LactD, and LactE, the frozen stocks of each lineage, sampled at intermediate points during their evolution, were screened for the appearance of each mutation found in the endpoint by Sanger sequencing of PCR-amplified mutation regions (Figure 4; Additional data file 5). A SNP was considered present if the dominant signal peak from Sanger sequencing indicated the mutation, although SNPs were at times observed at lower levels in the population as non-dominant peaks in the sequencing trace.

One may reasonably expect to see stepwise increases in growth rate during adaptation as additional mutations are acquired. However, in LactA, LactC, and LactD, mutations tend to be detected in groups, rather than step-wise, in time points corresponding to the end of an approximately 2-week period of rapid adaptation (day 14 or 19). The sudden appearance of multiple mutations may be indicative of competition within the population between different mutants during the period of rapid adaptation, but a countless number of other interpretations are possible. While other strains experienced a period of rapid adaptation, LactE had a gradual evolutionary trajectory, with mutations appearing more slowly over the 60 days of adaptation, and in a step-wise fashion. Mutations in *yjbM *and *acpP *were not yet dominant in the sequence traces of these screens, suggesting they were not yet fixed in the LactE population at day 60.

For mutations that were not found to fix in the population, we screened several individual colonies of the endpoint population for presence of the unfixed mutation (Additional data file 5). Of 12 LactE colonies at day 60, 4 had the *yjbM *mutation and the *acpP *mutation. The remaining eight colonies had neither mutation. The appearance of new mutations at day 60 may suggest adaptive evolution was incomplete in this strain, although a further 10 days of adaptive evolution failed to result in a significant increase in growth rate [12]. In addition to these two mutations, an *atoS *mutation detected using whole genome sequencing of LactD was not detected in the day 60 population of LactD. Further sequencing of this gene in the LactD endpoint using 12 additional colonies revealed no detectable mutation in *atoS *within the population. Because isolated single colonies from a mixed population were sequenced by Solexa and CGS, this mutation may have been unique to that colony. Alternatively, the mutation was present at a very low frequency in the adaptive endpoint culture.

### Fitness contribution of acquired mutations

Site-directed mutagenesis was used to create single and multiple mutants to directly assess the contributions of mutations individually and in combination on the phenotype of adaptive endpoint strains [10]. We created a subset of possible individual and combination mutants drawn from mutations discovered in the LactA, LactC, LactD, and LactE endpoints. We attempted site-directed mutagenesis for all SNPs and indels found in the LactA, LactC, LactD, and LactE endpoint strains, yet were unable to isolate mutants for every observed mutation due to difficulties at the cloning step of gene gorging or in finding successful recombinants. Of the four strains attempted, we were able to create a mutant with all discovered mutations for LactC only.

The growth rate recoveries of the constructed mutants in lactate M9 minimal media are shown in Table 2. A 0% growth rate recovery indicates the mutant grows no faster than the wild-type, pre-evolved strain in lactate minimal media while a mutant with 100% growth rate recovery grows at the same rate as its respective adaptive endpoint. We found that most single mutations produced from 1 to 26% growth rate recovery. The single exception was the LactD *kdtA *mutation, which was auxotrophic for amino acids, requiring supplementation of the M9 glycerol minimal media in order to grow. Addition of other mutations removed this requirement, and, in general, combinations of mutations resulted in at least approximately additive increases to the growth rate. In some cases, such as the LactC '*cya *+ *infC *+ *rph*' and '*relA *+ *ppsA*' mutant reconstructions, the addition of a mutation resulted in an increase in growth rate that was significantly greater than the additive increase in growth rate expected from the sum of individual mutations. Such observations suggest positive epistatic relationships between the mutations, which are essentially synergistic contributions of groups of mutations to fitness. Positive epistatic interactions between mutations acquired by the same strain during adaptive evolution have previously been confirmed by highly sensitive competition experiments [25].

**Table 2:** Growth rate recovery of site-directed mutants

Strain	Mutations	Growth rate (± SD)	Known mutations present	Recovery
Wild type		0.23 ± 0.02	-	-
				
LactA	*crp*	0.29 ± 0.02	1/3	26%
	Endpoint	0.47 ± 0.03	-	-
				
LactC	*rph*	0.27 ± 0.002	1/3	17%
	*cya*	0.26 ± 0.03	1/3	13%
	*infC*	0.26 ± 0.003	1/3	12%
	*cya *+ *infC*	0.31 ± 0.01	2/3	39%
	*cya *+ *infC *+ *rph*	0.40 ± 0.02	3/3	82%
	Endpoint	0.44 ± 0.01	-	-
				
LactD	*kdtA*	No growth	1/7	No growth
	*atoS*	0.24 ± 0.01	1/7	2%
	*ppsA*	0.23 ± 0.01	1/7	1%
	*relA*	0.28 ± 0.01	1/7	19%
	*rho*	0.25 ± 0.003	1/7	9%
	*relA *+ *ppsA*	0.33 ± 0.01	2/7	38%
	*kdtA *+ *ppsA*	0.27 ± 0.02	2/7	15%
	*kdtA *+ *ppsA *+ *atoS*	0.28 ± 0.01	3/7	21%
	*kdtA *+ *ppsA *+ *atoS *+ *rhoO*	0.34 ± 0.03	4/7	42%
	*kdtA *+ *ppsA *+ *atoS *+ *rho *+ *relA*	0.39 ± 0.01	5/7	64%
	Endpoint	0.48 ± 0.05	-	-
				
LactE	*yjbM*	0.23 ± 0.02	1/7	1%
	*ppsA*	0.25 ± 0.02	1/7	10%
	*crp*	0.27 ± 0.02	1/7	17%
	*ppsA *+ *crp*	0.28 ± 0.03	2/7	24%
	*ppsA *+ *crp *+ *yjbM*	0.31 ± 0.04	3/7	37%
	Endpoint	0.43 ± 0.02	-	-

To determine the causality of the observed mutations, site-directed mutagenesis was used to place mutations individually and in combination into a wild-type (MG1655) background. Average growth rate measurements of strains grown at 30°C in lactate M9 minimal media are shown. Growth rate recovery is defined as the difference in growth rate between the mutant and wild type, divided by the difference in growth rate between the respective endpoint strain and wild type. The *kdtA *single mutant was unable to grow without amino acid supplementation.

Mutations of genes that are frequently found to mutate in the adaptive condition are often the most beneficial [10,11]. It was therefore unexpected that the *rph*-*pyrE *single mutant induced only an approximately 15% growth advantage since the mutation was found in more than half of the adaptive endpoint strains. However, the addition of the *rph*-*pyrE *mutation to a LactC double mutant increased the growth rate recovery by approximately 30%, suggesting that the *rph*-*pyrE *mutation may have positive epistatic interactions with co-acquired mutations. The *rph*-*pyrE *mutation may be commonly found in the endpoints because it has positive epistatic interactions with a variety of mutational backgrounds. Alternatively, the appearance of the same 82-bp deletion in several endpoint strains suggests that this particular deletion is prone to occur in MG1655, and the mutation may frequently be found in endpoint strains simply because it gives some benefit for growth in lactate minimal media and arises frequently in the population.

## Conclusions

The affordability and capability of DNA sequencing platforms has allowed the determination of the genetic basis of adaptive evolution in bacteria. This technology is new, and only a handful of such studies have been reported. Because the parameters of adaptive evolution (such as mutation number, types of genes mutated, distributions of mutation fitness effects, and so on) vary with condition, more work is needed to reach general conclusions regarding genetic changes occurring after short-term laboratory adaptations of bacteria. In terms of experimental design, one clear lesson from the work described within is that the number and types of mutations even between replicates may have substantial variance and many replicates may, therefore, be needed to determine the variance of adaptive outcomes in a single condition and thus draw meaningful comparisons between conditions. We anticipate fundamental patterns of adaptation will become apparent as the increasing ease of these adaptive evolution sequencing studies leads to more published studies in the near future, and we hope this work will be of use to those designing such experiments.

## Materials and methods

### DNA and PCR

DNA extraction was performed using DNAeasy spin columns (Qiagen Germantown, MD, USA). PCR was performed using HotStar Taq Mastermix (Qiagen). Sanger sequencing was performed by EtonBio (San Diego, CA, USA). Primers used are listed in Additional data file 6.

### Adaptive evolutions

*E. coli *K-12 MG1655 (ATCC #47076; LactF to LactK) or a derivative (WT-A or BOP265 [10]) with identical growth rate (LactA to LactE) was used to inoculate starting cultures grown in 2 g/L L-lactate M9 minimal medium. Adaptive evolutions were carried out as previously described [10]. Serial passage was carried out for 60 days (LactA to LactE) or from 45 to 50 days (LactF to LactK; at least 700 generations) until growth rate remained stable from day to day. Single colonies (clones) of the endpoints designated LactA-1, LactB-1, and so on were isolated for sequencing by Nimblegen and Solexa.

### Nimblegen resequencing

Genomic DNA from the endpoint clones was extracted, concentrated by ethanol precipitation, and sent to Nimblegen Systems (Reykjavík, Iceland) for comparative genome sequencing [5] using *E. coli *K-12 MG1655 (ATC #47076) as the reference strain. Primers were designed to amplify approximately 600 bases around the reported SNP for PCR followed by verification of the reported SNP by Sanger sequencing.

### Solexa resequencing

Genomic DNA (5 μg) isolated from single colonies of the endpoint strains was used to generate the genomic DNA library using the Illumina genomic DNA library generation kit following the manufacturer's protocol (Illumina Inc., San Diego, CA, USA). Briefly, bacterial genomic DNA was fragmented by nebulization. The ends of fragmented DNA were repaired by T4 DNA polymerase, Klenow DNA polymerase, and T4 polynucleotide kinase. The Klenow exo minus enzyme was then used to add an 'A' base to the 3' end of the DNA fragments. After the ligation of the adapters to the ends of the DNA fragments, the ligated DNA fragments were subjected to 2% 1× TAE agarose gel electrophoresis. DNA fragments ranging from 150 to 300 bp were recovered from the gel and purified using the Qiagen mini gel purification kit. Finally, the adapter-modified DNA fragments were enriched by PCR. The final concentration of the genomic DNA library was determined by Nano drop and validated by running 2% 1× TAE agarose gel electrophoresis. A 4 pM genomic DNA library was used to generate the cluster on the Flowcell following the manufacturer's protocol. The genomic sequencing primer v2 was used for all DNA sequencing. A 36 cycle sequencing run was carried out using the Illumina 1G analyzer following the manufacturer's protocol for LactA to LactE. LactB and LactD were later rerun on a 2G analyzer along with LactF to LactK.

### Genome sequence assembly and polymorphism identification

The Solexa output for each resquencing run was first curated to remove any sequences containing a '.' (period) indicating lack of a base call. We then used MosaikAligner (MP Stromberg, GT Marth, unpublished data) to iteratively align reads to the *E. coli *reference sequence (GI:48994873), where in each iteration a limit was placed on the allowed number of alignment mismatches. This limit was increased from 0 to 5, and unaligned reads were used as input to the next iteration, which had a more lenient mismatch limit. An in-house script (available upon request) was then used to compile the read alignments into a nucleotide-resolution alignment profile. Consistency and coverage were then assessed to identify likely polymorphic locations. Locations at which coverage was greater than 10× and for which indels were observed or the count of a SNP was greater than twice the count of the matched reference sequence nucleotide were considered to be likely polymorphic locations.

False negative rates were determined for this sequencing method by polymorphism identification using an *E. coli *reference sequence that had 1,000 SNPs, deletions, and insertions added at random, known locations. Insertion sizes were randomly and uniformly distributed between 1 and 4 bp and deletions were between 1 and 99 bp. Mutations were not permitted to overlap. Detection rates of SNPs, deletions, and insertions were determined separately by counting the fraction of each type of mutation that was marked as polymorphic by the above script when sequence data from an endpoint were mapped to the mutated reference genome.

### Site-directed mutagenesis

Mutagenesis was performed using a scarless method known as gene gorging [26]. The procedure was performed as described in the supplementary methods of [10].

### Growth rates

Growth rate experiments were performed by measuring the optical density at 600 nm (OD) of triplicate cultures over several time points in which 0.05 < OD < 0.30. Growth conditions used were identical to the conditions used for adaptive evolution, except that flasks were placed in a 30°C water bath instead of the 30°C air incubator used for adaptive evolution. Growth rate was defined as the slope of the linear best-fit line through a plot of ln(OD) versus time (hours).

### Allele frequency estimation

Ten to twelve clones were randomly selected from M9-lactate agar plates inoculated with frozen stocks of the day 60 adaptive evolution culture. A 200- to 300-bp region surrounding each mutation was amplified from extracted DNA by PCR and Sanger sequenced to determine its presence in each clone.

### Allele appearance estimation

The approximate time point that each mutation fixed in its relevant population was estimated by screening the frozen stocks of culture saved at intermediate time points during each evolution to lactate. The predominant presence or absence of each mutation at a time point was determined by PCR of the 200- to 300-bp region surrounding the mutation, followed by Sanger sequencing.

## Abbreviations

CGS: Comparative Genome Sequencing; indel: insertion or deletion mutation; OD: optical density at 600 nm; SNP: single nucleotide polymorphism.

## Authors' contributions

TMC performed the LactF to LactK adaptive evolutions, confirmed mutations reported by Solexa, assisted with gene gorging, and measured growth rates. ARJ confirmed mutations reported by Nimblegen and created the majority of the gene gorging mutants. MKA estimated the time of appearance of the mutant alleles and their frequency in the endpoints and edited the manuscript. CLB performed the mapping of Solexa reads to the *E. coli *genome sequence. YG and BX sequenced the endpoints using Solexa and performed an early mapping of the reads to the genome. BØP, ARJ, TMC, MKA, CLB, and YG conceived of experiments and wrote the manuscript.

## Additional data files

The following additional data are available with the online version of this paper: an Excel table listing mutations reported for LactA, LactB, LactC, LactD, and LactE strains using Nimblegen CGS arrays (Additional data file 1); an Excel table listing mutations reported for all strains using Solexa sequencing (Additional data file 2); an Excel table showing the false negative rate of our mutation detection algorithm using a reference sequence genome with 'mutations' inserted at known locations (Additional data file 3); an Excel table listing regulons enriched for differential expression in LactA, LactB, LactC, LactD, and LactE strains (Additional data file 4); an Excel table listing the presence or absence of mutations at time points or in colonies, as used for determination of mutation trajectory and population mutation penetration (Additional data file 5); an Excel table listing primers used in this study (Additional data file 6).

## Supplementary Material

Additional data file 1 Mutations reported for LactA, LactB, LactC, LactD, and LactE strains using Nimblegen CGS arrays. Click here for file

Additional data file 2 Mutations reported for all strains using Solexa sequencing. Click here for file

Additional data file 3 False negative rate of our mutation detection algorithm using a reference sequence genome with 'mutations' inserted at known locations. Click here for file

Additional data file 4 Regulons enriched for differential expression in LactA, LactB, LactC, LactD, and LactE strains. Click here for file

Additional data file 5 The presence and absence of mutations at time points or in colonies were used for determination of mutation trajectory and population mutation penetration. Click here for file

Additional data file 6 Primers used in this study. Click here for file
